# Alterations in dendrite and spine morphology of cortical pyramidal neurons in DISC1-binding zinc finger protein (DBZ) knockout mice

**DOI:** 10.3389/fnana.2015.00052

**Published:** 2015-04-30

**Authors:** Yoshihisa Koyama, Tsuyoshi Hattori, Tomoki Nishida, Osamu Hori, Masaya Tohyama

**Affiliations:** ^1^Department of Anatomy and Neuroscience, Graduate School of Medicine, Osaka UniversitySuita, Osaka, Japan; ^2^Department of Neuroanatomy, Kanazawa University Graduate School of Medical SciencesKanazawa, Ishikawa, Japan; ^3^Department of Child Development and Molecular Brain Science, United Graduate School of Child Development, Osaka University, Kanazawa University, Hamamatsu University School of Medicine, Chiba University and University of Fukui SuitaOsaka, Japan; ^4^Research Center for Ultra-High Voltage Electron Microscopy, Osaka UniversitySuita, Osaka, Japan; ^5^Division of Molecular Brain Science, Research Institute of Traditional Asian Medicine, Kinki UniversityOsaka-sayama, Osaka, Japan; ^6^Osaka Prefectural Hospital OrganizationOsaka, Japan

**Keywords:** pyramidal neuron, dendrite, spine, synapse, PSD, DBZ, DISC1

## Abstract

Dendrite and dendritic spine formation are crucial for proper brain function. DISC1-binding zinc finger protein (DBZ) was first identified as a Disrupted-In-Schizophrenia1 (DISC1) binding partner. DBZ is highly expressed in the cerebral cortex of developing and adult rodents and is involved in neurite formation, cell positioning, and the development of interneurons and oligodendrocytes. The functional roles of DBZ in postnatal brain remain unknown; thus we investigated cortical pyramidal neuron morphology in DBZ knockout (KO) mice. Morphological analyses by Golgi staining alone in DBZ KO mice revealed decreased dendritic arborization, increased spine density. A morphological analysis of the spines revealed markedly increased numbers of thin spines. To investigate whole spine structure in detail, electron tomographic analysis using ultra-high voltage electron microscopy (UHVEM) combined with Golgi staining was performed. Tomograms and three-dimensional models of spines revealed that the spines of DBZ KO mice exhibited two types of characteristic morphology, filopodia-like spines and abnormal thin-necked spines having an extremely thin spine neck. Moreover, conventional electron microscopy revealed significantly decreased number of postsynaptic densities (PSDs) in spines of DBZ KO mice. In conclusion, DBZ deficiency impairs the morphogenesis of dendrites and spines in cortical pyramidal neurons.

## Introduction

Dendritic spines, which were first described by Ramón y Cajal are specialized structures on neuronal processes where the majority of excitatory synapses are localized (DeFelipe, 2015). Dendritic spines are highly dynamic during development and in the mature central nervous system and their morphology highly correlate with their functional properties. Altered dendritic spine density and morphology are associated with neurodevelopmental disorders such as fragile-X syndrome and Rett syndrome, which are monogenetic disorders that have a significant phenotypic overlap with Autism Spectrum Disorders (ASD; Hinton et al., 1991; Comery et al., 1997; Irwin et al., 2000, 2001). Therefore, proper spine formation is necessary for normal brain functions.

DBZ (DISC1-binding zinc finger protein) is an interacting partner of Disrupted-in-schizophrenia 1 (DISC1; Hattori et al., 2007). *DISC1* is a gene associated with major mental illnesses such as schizophrenia, mood disorders, autism and Asperger syndrome (Blackwood and Muir, 2004; Cannon et al., 2005; Kilpinen et al., 2008; Szeszko et al., 2008; Sprooten et al., 2011). Several genetic studies suggest that the DBZ gene is associated with schizophrenia and bipolar disorder (Liu et al., 2003; Segurado et al., 2003; Marcheco-Teruel et al., 2006; Moens et al., 2011). DBZ (also known as KIAA0844, ZNF365 and Su48), which has a predicted C_2_H_2_-type zinc-finger motif and coiled-coil domains, is localized diffusely in the cytoplasm and in the centrosome of cultured cells and neurons. Inhibition of the DISC1 and DBZ interaction causes impaired neurite outgrowth in PC12 cells and primary hippocampal neurons (Gianfrancesco et al., 2003). Furthermore, Su48 has been identified as a centrosomal protein essential for cell division. Overexpression of a mutant form of DBZ/Su48 *in vitro* disrupts the localization of γ-tubulin to the centrosome (Hirohashi et al., 2006; Wang et al., 2006).

DBZ mRNA is limitedly expressed in the central nervous system. An *in situ* hybridization study of the adult rat brain revealed strong expression of DBZ mRNA in the cortex and hippocampus (Hattori et al., 2007). In the developing cerebral cortex, DBZ mRNA expression starts on embryonic day 12.5–14.5 (E12.5–14.5) and expression levels gradually increase throughout the prenatal period. A recent study revealed that DBZ, together with DISC1, regulates cell positioning and neurite development by interfering with Ndel1 from disproportionate phosphorylation, which is critical for appropriate anterograde transport of the DISC1-complex (Okamoto et al., 2015). In postnatal brain DBZ mRNA expression increases until 6 weeks of age and maintains a high expression level thereafter (unpublished data). However, the importance of DBZ in a postnatal brain has not been fully elucidated.

In the present study, to clarify roles of DBZ in dendrite and spine formation of cortical pyramidal neurons, we investigated the morphology of spines and dendrites of pyramidal neurons in DBZ KNOCKOUT (KO) mice by Golgi staining. To obtain precise and detailed profiles of the spine morphology, we combined electron tomography with ultra-high voltage electron microscopy (UHVEM) with Golgi staining (Koyama et al., 2013b). In addition, to investigate effect of DBZ deficiency on synaptic formation, we analyzed the number of Postsynaptic densities (PSDs) in DBZ KO mice using transmission electron microscopy (TEM).

## Materials and Methods

### Animals

Twelve DBZ KO mice (male) and 12 of their wild type (WT) littermates (male) were used in experiments. Both groups of mice were aged 12 weeks. They were maintained under constant temperature with a 14-h/10-h light/dark cycle, and were given free access to water and rodent chow. The animal ethics committee of Osaka University approved all experimental procedures in accordance with the National Institute of Health (NIH) Guide for the Care and Use of Laboratory Animals. All efforts were made to minimize the number of animals used and to reduce their discomfort.

### Golgi Staining Procedure

Male mice (4 WT and 4 DBZ KO) aged 12 weeks were used. Brains were removed and Golgi-Cox staining was performed using an FD Rapid GolgiStain Kit (FD NeuroTechnologies, Ellicott City, MD, USA) according to the manufacturer’s instructions. Unfixed brain samples were processed as previously reported (Koyama et al., 2013a). The coronal brain blocks were immersed in a solution of equal parts Solution A and B at room temperature for 2 weeks and then soaked in Solution C at 4°C for 48 h. After freezing with dry-ice powder, the brain samples were sliced into 250 μm pieces at −22°C using a cryostat microtome. Each frozen section was mounted with Solution C on a 0.5% gelatin-coated glass slide. Slides were allowed to dry naturally at room temperature and were then stained with a mixture (Solution D: Solution E: DW; 1:1:2) for 5 min, after which the slides were rinsed in distilled water twice for 4 min each. Slides were dehydrated in an ascending ethanol series and then sealed with Entellan (Merck, Darmstadt, Germany) through xylene. The preparations were observed in detail with a BZ-9000 digital microscope (Keyence Corporation, Osaka, Japan).

### Parameter Analysis using Golgi-Stained Neurons

Camera-lucida drawings of cortical pyramidal neurons were obtained as previously reported (Koyama et al., 2013a). For parameter analyses, 16 pyramidal neurons in the somatosensory cortex of each mouse were randomly selected for assessment. As measurements for comparison analysis of dendrites and spines of pyramidal cells, we determined the number of primary dendrites arising from the soma and secondary and subsequent branch points, total dendrites and apical dendrite lengths, and the diameter of dendrites (diameter at the region where the apical dendrite arose from the soma) in each cell using a digital microscope with a 100× objective and ImageJ 1.44p software (NIH). We also determined spine density (mean numbers of spines per micrometer), spine head diameter and spine length (length from the tip to the point where the spine met the dendritic shaft). We further counted the number of spines in classified groups, a mushroom type (diameter of the spine head was larger than that of the spine neck), thin type (diameter of the spine head was equal to that of the spine neck), stubby spines (no neck), and ramified spines (two heads). For spine analyses, a total of 139 spines from 16 independent pyramidal neurons of DBZ KO mice were compared with those of 128 spines from 16 neurons of WT mice. To evaluate dendrite formation by pyramidal neurons, the dendritic arbors of each pyramidal cell were quantified based on the method described by Sholl (Sholl, 1953). A series of concentric rings with 16-mm-equivalent intervals were centered on the cell soma. The number of dendrites intersecting each consecutive ring was counted, yielding the number of dendrite intersections at set distances from the soma. The number of dendrites arising from the soma of pyramidal neurons was counted.

### Electron Tomography by Ultra-High Voltage Electron Microscopy (UHVEM)

Golgi stained samples for UHVEM were prepared as previously reported (Koyama et al., 2013b). After rinsing with 0.1 M phosphate buffer (PB; pH 7.4), the samples were fixed with 1% glutaraldehyde (GA; TAAB Laboratories Equipment Ltd., Aldermaston, England) in 0.1 M PB at 4°C overnight. Slides were washed in 0.1 M PB several times and then refixed with 1% OsO_4_ (TAAB) in 0.1 M PB for 1 h. Slides were then thoroughly washed in distilled water, followed by dehydration in an ascending ethanol series. Finally, the samples were embedded in epoxy resin (Quetol-812 set; Nisshin EM Corporation, Tokyo, Japan) through propylene oxide (Wako Pure Chemical Industries, Ltd., Osaka, Japan). The 4-μm-thick sections were mounted on formvar-coated 75-mesh Cu grids (Okenshoji Co., Ltd., Tokyo, Japan). Colloidal gold particles (60 nm diameter) were deposited on both sides of the sections for use as fiducail markers. After carbon coating (JEE-420; JEOL, Tokyo, Japan), sections were observed using UHVEM operating at 2 MeV (H-3000; Hitachi, Tokyo, Japan), and images recorded with a 4096 × 4096 pixel slow scan CCD camera (TVIPS, Gauting, Germany) at 4000× magnification. Single axis tilt series were recorded with an angular range of −60° to +60° in 2° increments. Tilt series were aligned using the gold particles and tomograms were reconstructed by weighted back-projection using the eTomo program in the IMOD software package (Kremer et al., 1996). The three-dimensional (3D) model of the dendritic spine structures was traced using drawing tools in IMOD. A total of 36 spines from DBZ KO mice and 24 spines from WT mice were analyzed.

### Transmission Electron Microscopy (TEM)

After fixation with 2% paraformaldehyde (PFA) and 2.5% GA in 0.1 M PB, brains were cut at 50 μm-thickness using Vibrating Blade Microtome (VT1000s; Leica., Wetzlar, Germany) and processed as previously described (Koyama et al., 2013b). The samples were refixed with 1% OsO_4_ in 0.1 M PB and embedded in epoxy resins. The 80-nm-thick ultrathin sections were mounted on the formvar-coated one-hole Cu grids (Nisshin EM Corporation) and stained with uranyl acetate and lead acetate. For analysis of PSDs were identified by tracing dendrites of pyramidal neurons using pictures of serial ultrathin sections obtained by TEM (H-7650; Hitachi, Tokyo, Japan). The number of PSDs per a spine of a synaptic contact was determined by counting the number of electron dense areas at the edge of a dendritic spine. A total of 192 synaptic contacts from DBZ KO mice and 170 synaptic contacts from WT mice were analyzed.

### Statistical Analysis

The effect of genotype × the total number of intersections of pyramidal neurons was analyzed using a two-way ANOVA with repeated measures. For all experiments except the analysis of the total number of intersections, Student’s *t*-test was used to compare the differences between WT and DBZ KO mice. The results of statistical tests were considered significant at *p* < 0.05. Results are expressed as mean values ± SEM.

## Results

### Morphological Analysis of the Dendrites and Spines of Cortical Pyramidal Neurons in DBZ KO Mice using Golgi Staining Alone

**Figure 1:**
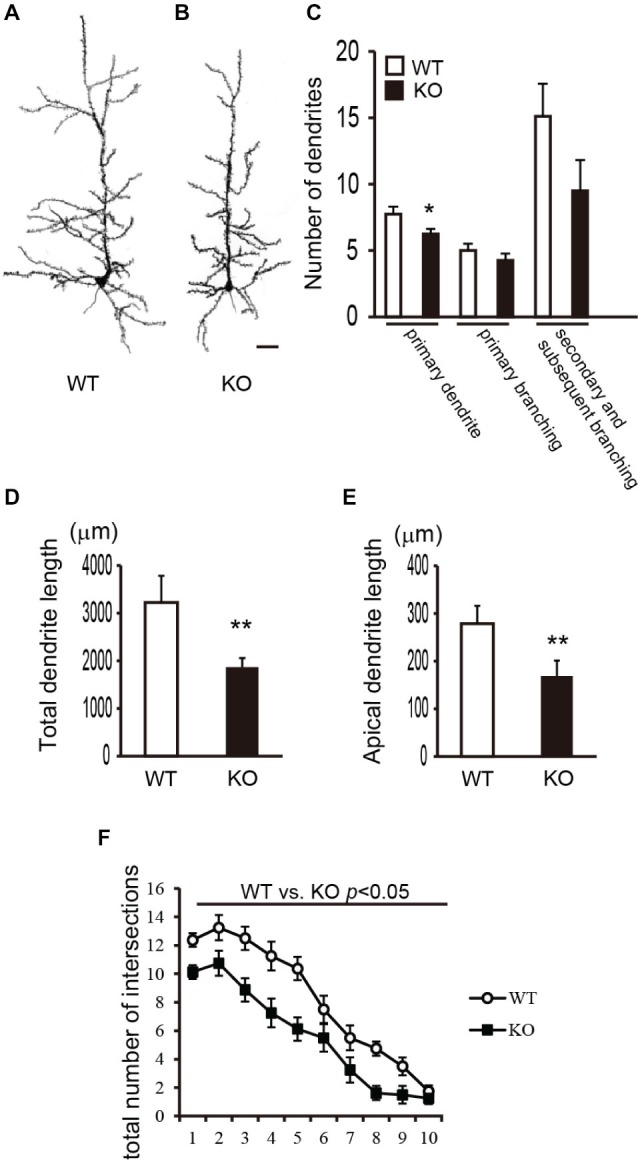
**Morphological analysis of dendrites of cortical pyramidal neurons in DBZ KNOCKOUT (KO) mice. (A,B)** Camera-Lucida tracing of Golgi-stained pyramidal neurons in the cerebral cortices of WILD TYPE (WT) **(A)** and DBZ KO mouse **(B)**. Scale bar, 50 μm. **(C)** Numbers of primary processes originating from the neuronal soma, the numbers of primary and secondary and subsequent branches were counted. **(D,E)** Total lengths of dendrites **(D)**, the length of apical dendrite **(E)** were measured. **(C–E)** data are expressed as means ± SEM of four animals per genotype. *p < 0.05 or **p < 0.01 vs. WT (Student’s *t*-test). **(F)** Total number of intersections of processes arising from the soma of pyramidal neurons in WT and DBZ KO mice was counted. Data are expressed as means ± SEM of four animals per genotype. *p < 0.01 vs. WT (two-way ANOVA).

To investigate the morphology of the dendrites and spines of cortical pyramidal neurons in DBZ KO mice, we performed Golgi-Cox staining and obtained camera lucida drawings of the pyramidal neurons in layer V of WT and DBZ KO mice. Representative images of pyramidal neurons in layer V are shown in Figure 1A (WT) and Figure 1B (DBZ KO). First, we counted the number of primary processes originating from neuronal soma including the apical dendrite and the number of primary or subsequent branches. In DBZ KO mice, the number of primary dendrites was significantly lower than that of WT mice (Figure 1C). Although not statistically significant, we also observed a trend towards decreased dendritic branching of pyramidal neurons in DBZ KO mice (Figure 1C). Second, we measured the total dendrite length and the length of apical dendrites using ImageJ. These lengths were significantly shorter in DBZ KO mice than in WT mice (Figures 1D,E). Third, to examine the complexity of dendritic arbor, we performed Sholl analysis and counted the number of intersections of dendrites with each concentric circle in a range from the soma to the distal ends of the dendrites. The number of intersections of dendrites was significantly decreased in DBZ KO mice, suggesting that the dendritic arborization of cortical neurons in DBZ KO mice was less complex than that in WT mice (Figure 1F).

**Figure 2:**
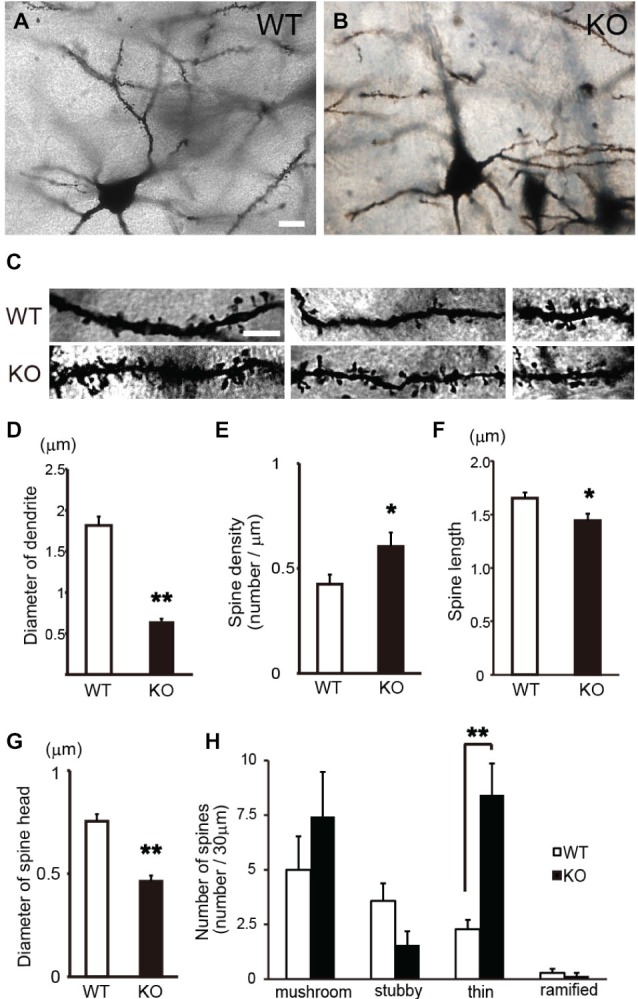
**Morphological analysis of spines of cortical pyramidal neurons in DBZ KO mice using Golgi staining alone. (A,B)** Images of Golgi-stained pyramidal neurons in the cerebral cortices of WT and DBZ KO mice at low magnification. Scale bar, 10 μm in **(A)** applying to **(B). (C)** Images of dendrites in WT and DBZ KO mice at high magnification. Scale bar, 5 μm in the left upper panel applying to all the panels in **(C). (D,E)** Diameter of dendrite and spine density were measured. Spine length **(F)** and diameter of spine head **(G)** were measured. **(H)** Spine morphology was classified to four types; mushroom spine, stubby spine, thin spine and ramified spine. The numbers of each spine per 30 μm dendritic segments were shown. All measured data are expressed as means ± SEM of four animals per genotype. *p < 0.05 or **p < 0.01 vs. WT (Student’s *t*-test).

Subsequently, to examine the morphological changes in the cortical pyramidal neurons of DBZ KO mice in detail, we performed a parametric analysis of the morphologies of the dendrites and spines in a high-power field in Golgi-stained pyramidal neurons (Figures 2A–C). In DBZ KO mice, thinner and uneven dendrites with increased number of spines were observed, when compared with those of WT mice (Figure 2C). Moreover, the diameter of the dendrites decreased (Figure 2D) and spine density increased (both significantly) in DBZ KO mice (Figure 2E). Furthermore, to evaluate spine morphology, we measured spine length and spine head diameter. The spine length of DBZ KO mice was slightly but significantly shorter than that of WT mice (Figure 2F). The spine head diameter of DBZ KO mice was also smaller than that of WT (Figure 2G). We classified the morphology of the spines in WT and DBZ KO mice according to the following four groups: mushroom spines, thin spines, stubby spines, and ramified spines. Significantly greater numbers of thin spines were observed in DBZ KO mice than in WT mice. Although not statistically significant, fewer stubby spines and ramified spines were observed in DBZ KO mice than in WT mice (Figure 2H). These results suggest that the formation of dendrites and spines is impaired in the pyramidal neurons of DBZ KO mice.

### Three-Dimensional Profiles of the Spines of Pyramidal Neurons in DBZ KO Mice Determined by UHVEM

**Figure 3:**
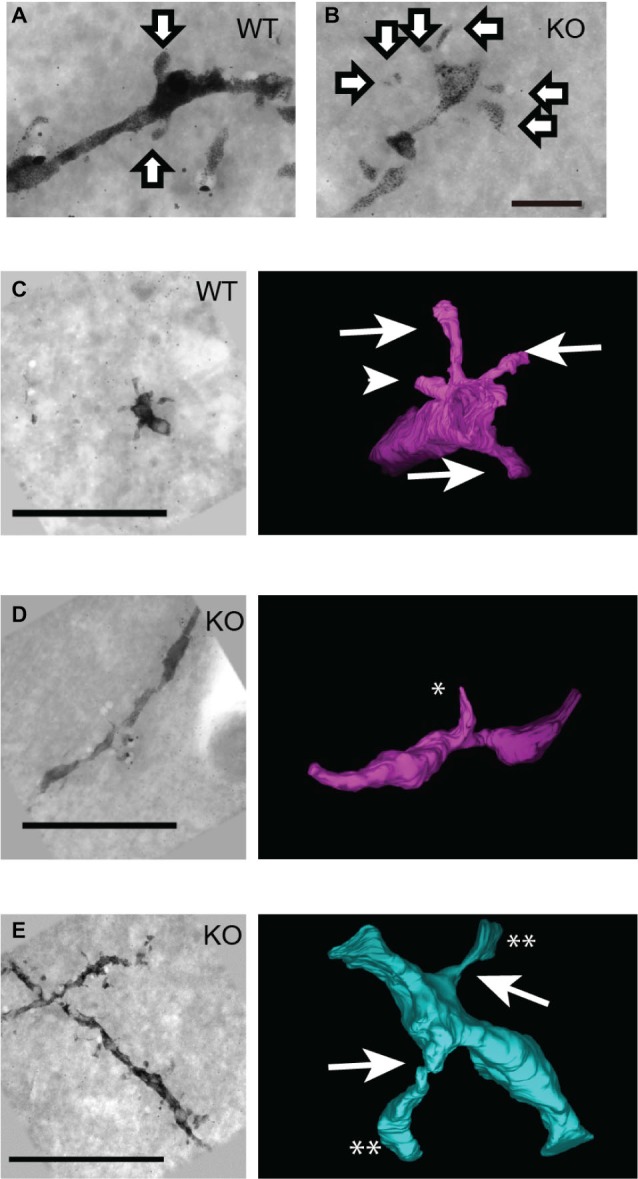
**Three-dimensional structural analysis of neural spines of DBZ KO mice using ultra-high voltage electron microscopy (UHVEM). (A,B)** Images of Golgi-stained spines of pyramidal neurons obtained with UHVEM in WT **(A)** and DBZ KO mice **(B)**. Arrows indicate spines of pyramidal neurons. Scale bar, 2 μm. **(C–E)** Pictures (left) and 3D reconstructions (right) of representative neural spines in WT **(C)** and KO mice **(D,E)**. Scale bars, 10 μm in **(C)** applying to **(D,E). (C)** Mushroom spines and stubby spine in WT mouse are indicated by arrows and arrowhead, respectively. **(D,E)** Images of immature spine, filopodia (**D**; an asterisk) and abnormal thin-necked spines (**E**; double asterisks) in DBZ KO mice. Arrows indicate extremely thin spine neck.

Golgi-staining analysis with light microscopy revealed an increased number of thin spines in DBZ KO mice. To obtain the whole profile of the spines in detail, we combined UHVEM with Golgi staining. UHVEM using 4 μm-thick sections provided 3D images of the spines as shown in Figures 3C–E. Consistent with Golgi staining results above, in WT mice, most of the observed spines were mushroom spines having a relatively large spine head and a spine neck (Figures 3A,C, arrows), and few stubby spines without a spine neck (Figure 3C, arrowhead). On the other hand, most of the spines in DBZ KO mice had a relatively longer and thinner spine neck (Figure 3B). The UHVEM results provided further detailed information on these spines found in DBZ KO mice. Judging from the 3D profiles of the spines observed in DBZ KO mice, they could be subdivided into two types: filopodia-like immature spines, having a thin and long spine neck and a sharp spine head (Figure 3D) or abnormal thin-necked spines, which had an extremely thin spine neck and a relatively small spine head (Figure 3E). The latter type outnumbered both the former and normal spines in DBZ KO mice.

### Analysis of PSDs of Pyramidal Neurons in DBZ KO Mice

**Figure 4:**
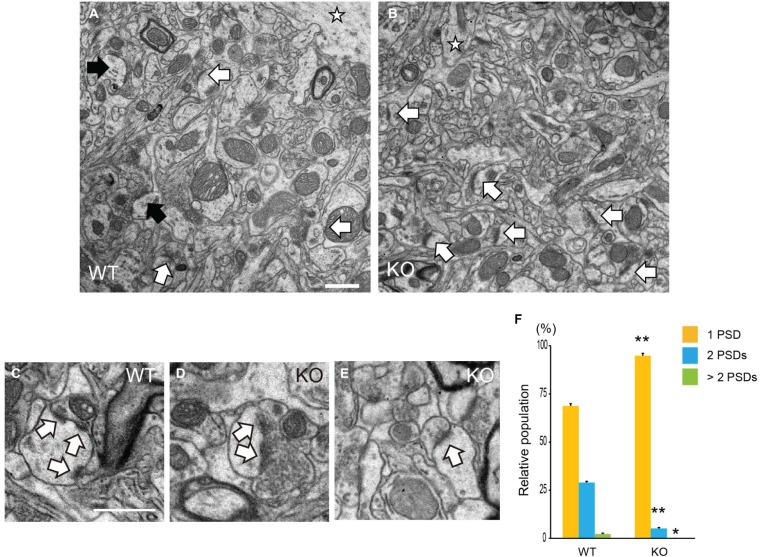
**Analysis of Postsynaptic densities (PSDs) in DBZ KO mice using transmission electron microscopy (TEM). (A–E)** TEM photomicrographs of synapses of cortical pyramidal neurons in WT **(A)** and DBZ KO mice **(B)**. Black and white arrows indicate spines with two PSDs and one PSD in a spine, respectively. Star indicates dendrite of pyramidal neuron. Scale bar, 1 μm in **(A)** applying to **(B). (C–E)** Representative images of three PSDs in a spine **(C)** in WT mouse and two PSDs **(D)** and one PSD **(E)** in a spine in DBZ KO mouse. Arrows indicate PSDs. Scale bar, 0.5 μm in **(C)** applying to **(D,E). (F)** Spines were classified into three groups, one PSD, two PSDs and more than three PSDs in a spine of a synaptic contact. The population of each spine to total number of spine contacts was calculated. All measured data are expressed as means ± SEM of four animals per genotype. *p < 0.05 or **p < 0.01 vs. WT (Student’s *t*-test).

Specific spine shapes are associated with distinct synapse morphologies and subcellular organelles, such as PSDs. PSD is attached under the surface of the spine membrane either at the top or side of the spine head, across from a vesicle-containing presynaptic axon. PSDs are observed by EM as an electron-dense region at the membrane of a dendritic spine. We observed the PSDs of pyramidal neurons in the cerebral cortices of DBZ KO mice by TEM. Using TEM images of the synapses of pyramidal neurons in WT and DBZ KO mice, we counted the number of PSD in a spine (a total of 170 and 192 synaptic contacts in WT and DBZ KO mice, respectively; Figures 4A,B). In DBZ KO mice, the number of PSDs in a spine was significantly decreased compared with that of WT mice. (WT vs. DBZ KO): one PSD: (68.8 ± 1.3% vs. 94.8 ± 0.9%); two PSDs: (28.8 ± 2.1% vs. 5.1 ± 0.9%); more than three PSDs (2.3 ± 0.9% vs. 0 ± 0%) (Figures 4C–F).

## Discussion

In the present study, observations using Golgi staining alone revealed that cortical pyramidal neurons of DBZ KO mice exhibited decreased dendritic arborization and increased numbers of spines. A morphological analysis of the spines revealed markedly increased numbers of thin spines. The subsequent UHVEM experiments revealed that the spines of DBZ KO mice exhibited two types of characteristic morphology, filopodia-like spines and abnormal thin-necked spines having an extremely thin spine neck. Finally, TEM demonstrated that DBZ KO pyramidal neurons exhibited decreased numbers of PSDs in the spines. These findings suggest that deficiency of the DBZ gene impaired proper formation of dendrites and spines in cortical pyramidal neurons.

In this study the length and the branching of dendrites in pyramidal neurons were altered in DBZ KO mice. This result is supported by a previous study indicating that the interaction between DBZ and DISC1 is involved in neurite outgrowth in rat primary culture neurons (Hattori et al., 2007). Moreover, in DBZ KO mice, neurite formation is also impaired in cortical interneurons and pyramidal neurons (Koyama et al., 2013a). A recent study showed that DBZ deficiency results in poor transportation of DISC1 and LIS1 to the neurite ends and hampers microtubule elongation (Okamoto et al., 2015). Microtubules not only provide structural support for dendrites, but also participate in dendrite outgrowth and branching (Georges et al., 2008). Therefore, impaired microtubule organization could alter the length and branching of dendrites in DBZ KO mice. A previous study demonstrated the presence of microtubules not only in dendritic shafts but also in dendritic spines, mainly in mushroom spines (Gu et al., 2008). Microtubules that penetrate dendritic spines are highly dynamic and contain the microtubule plus end tracking protein EB3 (Hu et al., 2008). Inactivation of EB3 by shRNA reduces dendritic spine formation (Gu et al., 2008). Thus, impaired microtubule organization in DBZ KO mice could also induce altered dendritic spine morphology. Furthermore, knockdown of DISC1 increases the size and number of spines in glutamate synapses (Hayashi-Takagi et al., 2010). It is possible that inappropriate transportation of DISC1 to dendritic spines caused by DBZ deficiency results in an increased number of spines and altered spine morphology.

Observations with Golgi staining alone revealed an increased number of thin spines in DBZ KO mice compared with that of WT mice. In the subsequent analysis using UHVEM, we found that these thin spines had a filopodia-like shape. In immature neuron, very thin and long dendritic protrusions are called dendritic filopodia. In mature neurons, these filopodia form each dendritic spines (Sekino et al., 2007). Therefore, a larger number of spines might be in a more immature state in the pyramidal neurons of DBZ KO mice than in those of WT mice. Interestingly, UHVEM also identified a characteristic spine shape with an extremely thin spine neck in DBZ KO mice. As these spines have larger spine head than neck, they were identified as mushroom spines by the observation with light microscopy. Observations using UHVEM and 3D tomography enabled detection of fine morphological changes in the spines of DBZ KO mice.

Spine head size positively correlates with synaptic strength, as the PSD dimensions are proportional to total spine volume (Spacek and Harris, 1997). Therefore, the decreased number of PSDs in spines of DBZ KO mice may result from the decreased volume of spine head and the increased number of thin spines. The PSD ranges in shape from a simple disc (macular PSD) or a perforated annulus (perforated PSD), to a highly irregular or segmented structure (Sorra and Harris, 2000). The decreased number of PSDs suggests decreased postsynaptic density area in DBZ KO mice. Thus, there might be more macular PSD and less perforated PSD at the synaptic contacts of DBZ KO mice. In excitatory neurons glutamate receptors aggregate in the PSDs of the spine, where glutamate receptors possess synaptic function (Matsuzaki et al., 2001). Since PSD dimensions correlate with the sensitivity of glutamic acid, excitatory neurotransmission might be altered in the synapses of DBZ KO mice. Furthermore, the morphology of the spine neck influences the kinetics of calcium which is inflowing through glutamate receptor of a spine. Thin spine necks prevent calcium within the spine head from entering into the dendrite (Majewska et al., 2000). Therefore, since DBZ KO mice exhibited thinner spine necks than WT mice, calcium signal transduction may be reduced in these spines. In spines, calcium/calmodulin-dependent protein kinase II (CAMKII) is activated in a calcium concentration-dependent manner and is involved in long-term potentiation (Colbran and Brown, 2004). Taken together, the altered morphology of the spine head and neck in DBZ KO mice might affect synaptic efficacy in cortical pyramidal neurons.

Immature spine morphology, such as the filopodia-like spines observed in DBZ KO mice, is reported in several animal models of neurological disorders. Transgenic mice expressing the exon 2-defective form of alpha thalassemia/mental retardation syndrome X-linked (ATRX) protein exhibit thin and long, filopodia-like spines. ATRX transgenic mouse shows impaired memory and cognitive function-related behavior (Shioda et al., 2011). In addition, FMRP (Fragile-X Mental Retardation Protein) KO mice, a model of Fragile-X syndrome, also exhibit a significant increase in the density of thin and long dendritic spines as well learning and memory deficits (Comery et al., 1997; Irwin et al., 2000; McKinney et al., 2005; Dölen et al., 2007). Therefore, the increased number of immature spines in DBZ KO mice might result in impaired learning, memory, and cognition. Behavioral and electrophysiological studies, are needed to clarify the effect of these morphological changes on physiological functions in DBZ KO mice. Therefore, further analysis of neuronal development using DBZ KO mice will help elucidate the mechanisms underlying the formation of neuronal networks and the pathology of psychiatric illnesses.

## Conflict of Interest Statement

The authors declare that the research was conducted in the absence of any commercial or financial relationships that could be construed as a potential conflict of interest.
